# Erroneous Classification and Coding as a Limitation for Big Data Analyses: Causes and Impacts Illustrated by the Diagnosis of Clavicle Injuries

**DOI:** 10.3390/diagnostics15020131

**Published:** 2025-01-08

**Authors:** Robert Raché, Lara-Sophie Claudé, Marcus Vollmer, Lyubomir Haralambiev, Denis Gümbel, Axel Ekkernkamp, Martin Jordan, Stefan Schulz-Drost, Mustafa Sinan Bakir

**Affiliations:** 1 Department of Orthopedics, Trauma Surgery and Rehabilitative Medicine, University Medicine Greifswald, Ferdinand-Sauerbruch-Straße, 17471 Greifswald, Germany; robert.rache@stud.uni-greifswald.de (R.R.); lara.claude@gmx.de (L.-S.C.); lyubomir.haralambiev@med.uni-greifswald.de (L.H.); denis.guembel@ukb.de (D.G.); ekkernkamp@ukb.de (A.E.); martin.jordan@med.uni-greifswald.de (M.J.); 2 Institute of Bioinformatics, University Medicine Greifswald, Felix-Hausdorff-Str. 8, 17475 Greifswald, Germany; marcus.vollmer@uni-greifswald.de; 3 Department of Trauma Surgery and Orthopedics, BG Hospital Unfallkrankenhaus Berlin gGmbH, Warener Straße 7, 12683 Berlin, Germany; 4 Department of Trauma and Orthopedic Surgery, University Hospital Erlangen, Krankenhausstr. 12, 91054 Erlangen, Germany; 5 Department of Trauma Surgery, Helios Hospital Schwerin, Wismarsche Str. 393-397, 19049 Schwerin, Germany

**Keywords:** big data analyses, AI-based analyses, misclassification, clavicle injuries, clavicle fractures

## Abstract

**Background/Objectives**: Clavicle injuries are common and seem to be frequently subject to diagnostic misclassification. The accurate identification of clavicle fractures is essential, particularly for registry and Big Data analyses. This study aims to assess the frequency of diagnostic errors in clavicle injury classifications. **Methods**: This retrospective study analyzed patient data from two Level 1 trauma centers, covering the period from 2008 to 2019. Included were cases with ICD-coded diagnoses of medial, midshaft, and lateral clavicle fractures, as well as sternoclavicular and acromioclavicular joint dislocations. Radiological images were re-evaluated, and discharge summaries, radiological reports, and billing codes were examined for diagnostic accuracy. **Results**: A total of 1503 patients were included, accounting for 1855 initial injury diagnoses. In contrast, 1846 were detected upon review. Initially, 14.4% of cases were coded as medial clavicle fractures, whereas only 5.2% were confirmed. The misclassification rate was 82.8% for initial medial fractures (*p* < 0.001), 42.5% for midshaft fractures (*p* < 0.001), and 34.2% for lateral fractures (*p* < 0.001). Billing codes and discharge summaries were the most error-prone categories, with error rates of 64% and 36% of all misclassified cases, respectively. Over three-quarters of the cases with discharge summary errors also exhibited errors in other categories, while billing errors co-occurred with other category errors in just over half of the cases (*p* < 0.001). The likelihood of radiological diagnostic error increased with the number of imaging modalities used, from 19.7% with a single modality to 30.5% with two and 40.7% with three. **Conclusions**: Our findings indicate that diagnostic misclassification of clavicle fractures is common, particularly between medial and midshaft fractures, often resulting from errors in multiple categories. Further prospective studies are needed, as accurate classification is foundational for the reliable application of Big Data and AI-based analyses in clinical research.

## 1. Introduction

Clavicle injuries are a common occurrence, with fractures being the most prevalent type [1,2,3]. According to the literature, the majority of these injuries are midshaft clavicle fractures (69%), followed by lateral fractures (28%), with medial clavicle fractures being the least common (3%) [4,5,6]. The treatment of clavicle fractures varies significantly depending on the location and nature of the injury [2,7,8,9]. Unlike midshaft clavicle fractures, for which standardized treatment algorithms and surgical procedures exist, there is no consensus on the management of medial clavicle fractures [6,10,11,12,13]. Medial clavicle fractures are complex and should be managed in specialized centers, with clear differentiation from midshaft clavicle fractures [10,11,14]. Recent Big Data analyses of routine data have indicated that medial clavicle fractures may be more frequent than previously assumed [3,10]. Conversely, recent studies have also suggested an increase in misclassification, particularly between medial and midshaft clavicle fractures [11]. However, the extent of this problem remains unexplored. The accurate classification of fracture location is crucial, as is the investigation of the issue of mislabeling and the underlying causes of these misclassifications.

The precise categorization of (clavicle) fractures is particularly important in the context of registry data analyses and Big Data studies [15,16]. These methodologies offer a valuable alternative for investigating rare injuries and conditions, especially when mono-institutional studies suffer from limited sample sizes [17,18]. Such studies have gained prominence, particularly in healthcare/health services research [16,19,20,21]. Inaccurately labeled diagnoses, particularly fractures, in large datasets could lead to erroneous conclusions when analyzed using artificial intelligence or machine learning [19,22,23]. This risk can be mitigated through correct labeling.

It is hypothesized that the misclassification of clavicle injuries is more prevalent than currently recognized, particularly between medial and midshaft clavicle fractures. This misclassification may arise due to various factors, including linguistic ambiguities in the documentation of fracture types, as well as possible insufficient attention to detail in the process of clinical coding practices. We also hypothesize that these misclassifications may arise from various sources, such as imaging diagnostics, discharge summaries, or billing processes/case management, and that the propagation of these errors is likely. This study primarily aims to investigate the frequency of misclassification in clavicle fractures, with a secondary objective of analyzing the errors concerning fracture location and their origins. Additionally, the study seeks to identify the influence of specific factors that may have an effect on the incidence of misclassification since we assume that the prevalence of such errors is likely to be higher in cases where clear distinctions between injury locations are challenging.

## 2. Materials and Methods

This study was designed as a bicentric, retrospective analysis. Patient-related data were collected from two Level 1 trauma centers, involving patients with clavicle-associated shoulder girdle injuries between 2008 and 2019. All data were derived from digital patient medical records. Inclusion criteria encompassed all patients with medial, midshaft, and lateral clavicle fractures (classified according to ICD-10 codes S42.01, S42.02, S42.03, representing a fracture in the medial, middle, and lateral third, respectively), as well as acromioclavicular and sternoclavicular joint dislocations (classified under ICD-10 codes S43.1 and S43.2) [24]. No age limitation was applied. Data sources included radiographic images (X-ray), computed tomography (CT), and magnetic resonance imaging (MRI) scans, all of which were re-evaluated and cross-checked for diagnostic accuracy using a rigorous double-review process. Moreover, all available radiological modalities for each case were utilized to ensure comprehensive assessments. In addition to the diagnostic imaging, discharge summaries, radiological reports, and coding of billing data were reviewed, focusing on the consistency of injury labels according to ICD-10 codes [24]. The cohort was selected by reviewing all patients treated for clavicle injuries during the specified period and then identifying those with complete diagnostic data for inclusion in the study. The verification process was conducted by a panel of three reviewers, with final diagnostic conclusions reached by consensus, ensuring agreement among senior expert trauma surgeons and ensuring that each case was accurately classified according to the true injury pattern.

Injuries could occur as isolated or combined injuries, allowing for multiple valid diagnoses within a single patient case. As a result, the total number of initial diagnostic labels (derived from CT, MRI, radiographs, discharge summaries, and billing codes) and the actual diagnoses (the truly present injuries) could differ. This discrepancy arose from the possibility of multiple injuries in a single patient, or from cases where fewer injuries were present than initially described, or where no actual injury was found. The primary exclusion criterion was the inability to verify cases due to unavailable data, such as missing radiographs from external pre-treatment, or insufficient accuracy/quality of radiological images and clinical documentation.

A label was classified as erroneous if an error was detected in at least one of the categories (discharge summary, radiological report, coding). An error was defined as the description of a diagnosis that was not actually present (false positive) or the omission of an actual diagnosis (false negative). All erroneous labels were categorized according to their nature (false positive, false negative) and analyzed to identify patterns or recurring issues in diagnostic classification. The number of erroneous categories was also analyzed. Further analysis included an investigation of potential factors influencing misclassification, with a focus on evaluating injury complexity (based on multi-fragmentary fracture morphology and radiating fracture lines in multiple fragments) and the type and number of radiological diagnostic methods performed.

Findings were reviewed, re-evaluated, and statistically analyzed based on the newly obtained and controlled data to evaluate the frequency and nature of diagnostic errors, as well as to identify factors influencing misclassification. Misclassification rates were calculated along with 95% Clopper–Pearson confidence intervals. The Chi-square test was chosen for its suitability in examining associations between categorical variables. Chi-square tests were employed for the comparison of proportions of errors between the variables of different injury types, diagnostic methods, and imaging modalities. Based on Chi-square tests, we also statistically analyzed influencing factors such as injury complexity and the number of radiological methods. A sufficiently large sample size in each category was proved and confirmed, as an assumption of the Chi-square test, to ensure the validity of the results. Statistical analyses were conducted using SPSS (IBM Inc., Armonk, NY, USA, Version 26) and R (Version 4.0.0) [25].

The study was registered with the German Clinical Trials Register (DRKS; DRKS00017016). Where required, patients provided written informed consent for their participation in the study. The study was conducted in accordance with the ethical standards of the 1964 Declaration of Helsinki and was approved by the relevant clinical ethics committee (Ethics Committee of the University Medicine Greifswald, BB 007/19).

## 3. Results

### 3.1. Patient Population and Data Overview

A total of 1597 patient cases involving one or more of the five clavicle-associated diagnoses were analyzed. After excluding 94 cases due to missing information, 1503 patients were included in the study (Table 1). Approximately one in four patients in the overall cohort was misclassified, with no significant difference observed between male and female patients (*p* = 0.432).

### 3.2. Error Frequency in Diagnosed Injuries

There was only a small discrepancy between the initially recorded diagnoses (*n* = 1855 injuries) and the actual confirmed diagnoses (*n* = 1846 injuries), resulting in a 0.49% reduction in detected injuries (Table 2). The most significant discrepancy was observed in medial clavicle fractures, which represented only 5.2% of the actual diagnoses compared to the initial 14.4%. The percentage breakdown highlights that while medial clavicle fractures were overrepresented in the initial diagnoses, midshaft clavicle fractures were significantly initially underreported.

### 3.3. Distribution of Errors by Fracture Location

**Figure 1:**
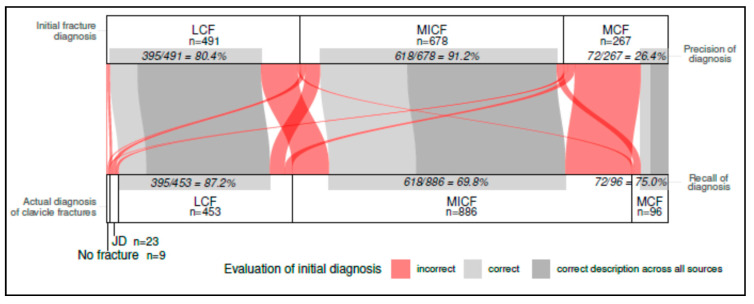
A comparison of the Initial Fracture Diagnosis according to the ICD 10 coding versus Corrected Coding and Actual Diagnosis of all Clavicle Fractures in a Sankey diagram. The precision rate and the recall rate of the respective diagnosis are presented. More than one option could be mentioned. The amount of completely correct descriptions across all sources is presented as well as the evaluation of correct vs. incorrect evaluated initial diagnosis. The data are presented in total numbers (*n*) and as percentage. MCF = medial clavicle fracture; MICF = midshaft clavicle fracture; LCF = lateral clavicle fracture; JD = joint dislocation (including sternoclavicular and acromioclavicular).

**Figure 2:**
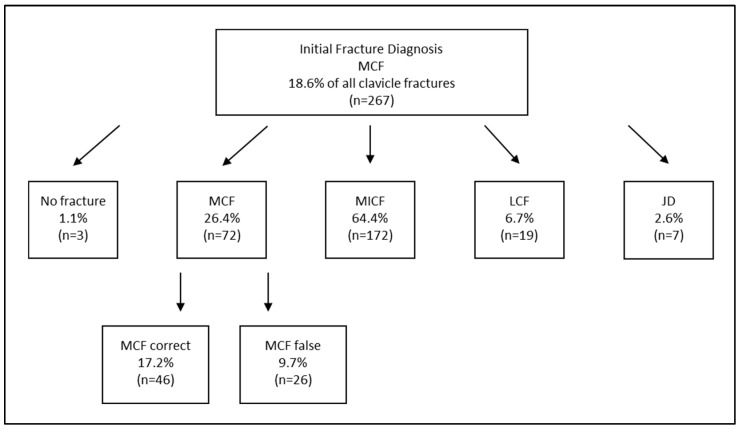
A comparison of the Initial Fracture Diagnosis according to the ICD 10 coding versus Corrected Coding and Actual Diagnosis of Medial Clavicle Fractures (=MCF). The numbers of the corrected actual diagnosis are presented as the percentage of the subareas initially coded MCF, MICF and LCF. More than one option could be mentioned wherefore percentage over 100% of the initial diagnoses is possible. The amount of completely correct descriptions across all sources is presented as the percentage of all actual medial clavicle fractures. MCF = medial clavicle fracture; MICF = midshaft clavicle fracture; LCF = lateral clavicle fracture; JD = joint dislocation (including sternoclavicular and acromioclavicular).

**Figure 3:**
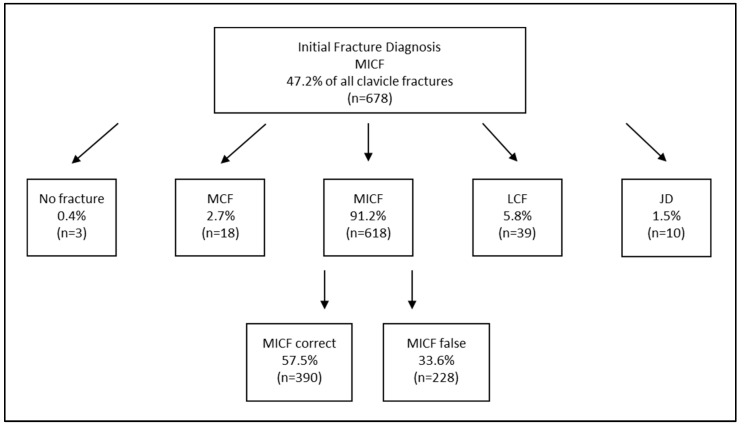
A comparison of the Initial Fracture Diagnosis according to the ICD 10 coding versus Corrected Coding and Actual Diagnosis of Midshaft Clavicle Fractures (=MICF). The numbers of the corrected actual diagnosis are presented as the percentage of the subareas initially coded MCF, MICF and LCF. More than one option could be mentioned wherefore percentage over 100% of the initial diagnoses is possible. The amount of completely correct descriptions across all sources is presented as the percentage of all actual midshaft clavicle fractures. MCF = medial clavicle fracture; MICF = midshaft clavicle fracture; LCF = lateral clavicle fracture; JD = joint dislocation (including sternoclavicular and acromioclavicular).

**Figure 4:**
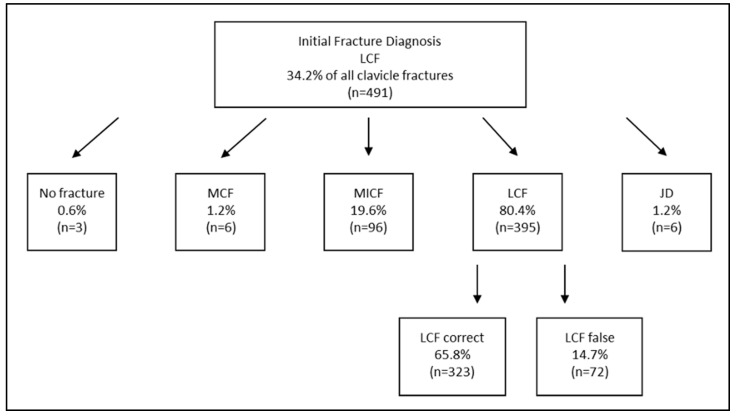
A comparison of the Initial Fracture Diagnosis according to the ICD 10 coding versus Corrected Coding and Actual Diagnosis of Lateral Clavicle Fractures (=LCF). The numbers of the corrected actual diagnosis are presented as the percentage of the subareas initially coded MCF, MICF and LCF. More than one option could be mentioned wherefore percentage over 100% of the initial diagnoses is possible. The amount of completely correct descriptions across all sources is presented as the percentage of all actual lateral clavicle fractures. MCF = medial clavicle fracture; MICF = midshaft clavicle fracture; LCF = lateral clavicle fracture; JD = joint dislocation (including sternoclavicular and acromioclavicular).

When focusing solely on clavicle fractures, the total number of confirmed fracture diagnoses decreased only from 1436 to 1435, representing almost no reduction (Table 3). However, substantial variation was observed among the diagnoses in relation to their localization across the subgroups (Figure 1). These differences were largely due to fractures being described differently across various sources (e.g., discharge summaries, imaging reports), yet corresponding to only a single actual diagnosis in some cases. For instance, out of *n* = 267 initial diagnoses of “medial clavicle fractures”, only 17.2% were fully correctly described in any category but not incorrectly in another, making it the least accurately coded clavicle-related injury (Figure 2). Conversely, more than half of the initial midshaft clavicle fractures were correctly coded, and nearly two-thirds of initial lateral clavicle fractures were accurately diagnosed (Figure 3 and Figure 4).

Among the initially diagnosed fractures, only 26.4% of the medial clavicle fractures (MCFs) were correctly classified as such, while 91.2% of midshaft clavicle fractures (MICF) and 80.4% of lateral clavicle fractures (LCFs) were confirmed as accurate diagnoses. However, even within these correctly diagnosed cases, a completely error-free description across all sources was only achieved in approximately two-thirds of cases for both actual MCF and MICF, and in over 80% of cases for actual LCF (Figure 1, Figure 2, Figure 3 and Figure 4). In conclusion, the findings demonstrate a significantly higher error rate for the initial coding of medial clavicle fractures (82.8%; 95% CI: [0.777; 0.871], *p* < 0.001), in contrast to midshaft fractures (42.5%; [0.387; 0.463], *p* < 0.001) and lateral fractures (34.2%; [0.300; 0.386], *p* < 0.001).

The data analysis revealed that actual medial and midshaft clavicle fractures were more frequently misdescribed (52.1% of all actual medial fractures were incorrectly described (95% CI: [41.64; 62.39]; *p* = 0.014), and 56.0% of midshaft fractures ([52.64; 59.28], *p* < 0.001)) compared to lateral fractures, which had a 28.7% misdiagnosis rate ([24.57; 33.10], *p* < 0.001). The most common specific error was the misclassification of a midshaft clavicle fracture as a medial fracture, representing 64.4% of the initial medial clavicle fracture diagnoses.

### 3.4. Sources of Error

**Figure 5:**
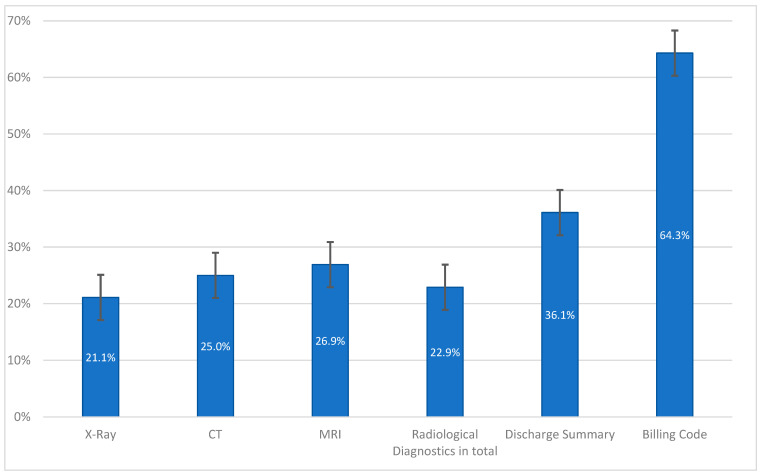
Error categories by percentage of misdiagnosis with 95% confidence interval. Single case may be misdiagnosed in multiple categories.

The analysis identified multiple error categories and shows the distribution of errors across different modalities, with the most error-prone areas being coding in billing codes, which had a 64.3% error rate, and discharge summaries, which had a 36.1% error rate (Figure 5).

**Figure 6:**
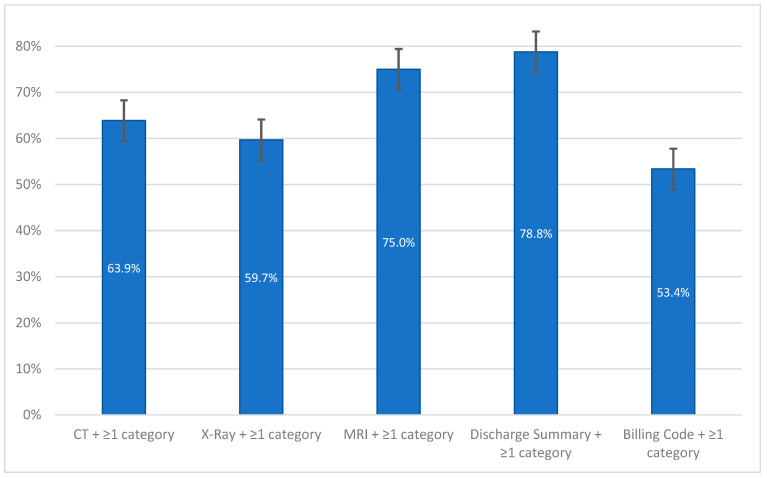
Multiplicity of errors in cases with misdiagnoses across different categories by percentage of misdiagnosis with 95% confidence interval. At least one additional error was present in these cases.

Further analysis revealed that most cases with errors in discharge summaries also had errors in other categories, whereas the amount differs significantly relating to the respective categories (Figure 6). Specifically, over 75% of cases with erroneous discharge summaries had errors in other areas, while just over half of the cases with billing errors were associated with additional coding errors (*p* < 0.001).

### 3.5. Impact of (Case) Complexity on Error Probability

Case complexity, assessed based on fracture morphology and the complexity of radiological diagnostics, was a significant factor in the likelihood of diagnostic errors. Fractures with complex patterns involving multiple clavicle regions (e.g., medial to lateral part) were more prone to errors, with a 13.5% error rate for fractures with multi-region involvement compared to a 2.4% error rate for simpler fractures (*p* < 0.001).

Additionally, the probability of errors increased with the use of more than one imaging modality (Table 4). For instance, only one in five cases using a single imaging modality was miscoded, while the error rate more than doubled with the use of three imaging modalities. The probability of diagnostic errors increased with the use of advanced three-dimensional imaging modalities such as computed tomography (CT) and magnetic resonance imaging (MRI). Fewer misclassifications were observed when only X-rays were used compared to cases where multiple imaging modalities were employed (*p* < 0.001).

## 4. Discussion

Our study demonstrates that a significant proportion of clavicle fractures in clinical practice are inaccurately diagnosed and coded. These errors are particularly frequent in distinguishing between medial and midshaft clavicle fractures. Such misclassifications pose a substantial risk for misinterpretation, especially in the context of Big Data analyses that rely on routine clinical data and associated coding.

### 4.1. Error Frequency in Relation to the Injury Entity

The initial distribution of injury classifications in our dataset deviated considerably from the literature. According to the literature, midshaft fractures account for more than two-thirds of all clavicle fractures, followed by lateral fractures at nearly one-third, with medial fractures being the least common, occurring in fewer than 5% of cases [5]. While the incidence of lateral clavicle fractures in our study was consistent with the literature, the frequency of midshaft fractures was notably lower, and medial clavicle fractures were overrepresented by more than threefold. After correcting for misclassifications, the incidence of all fracture types aligned more closely with the literature. Specifically, the frequency of midshaft clavicle fractures increased, while the incidence of medial fractures decreased to levels similar to those reported in prior studies [1,5]. This study confirms that diagnostic mislabeling is common, particularly for medial clavicle fractures. These fractures are frequently mislabeled, either as midshaft or lateral clavicle fractures, and the initial diagnosis of medial fractures is often incorrect. Special attention should be paid to avoid these errors, especially as the most common misclassification was the incorrect labeling of a true midshaft clavicle fracture as a medial fracture.

### 4.2. Consequences of Misclassification

The mislabeling of clavicle fractures has implications for both clinical practice and the interpretation of epidemiological data. Recent studies suggest an increasing incidence of medial clavicle fractures [3]. However, this trend may reflect a systematic error in diagnostic coding, as the mislabeling of these fractures cannot be excluded in Big Data analyses conducted on routine clinical datasets. The reliance on ICD-10 codes in such datasets introduces potential bias, especially since the data were anonymized and could not be cross-verified [23,26,27,28]. Even in retrospective studies using internal hospital data, diagnostic codes are often derived from billing data, which may lead to the over- or underrepresentation of certain injuries.

Thoroughness and accuracy in diagnosis are essential not only for Big Data analyses but also for the validity of many study designs. Misclassification or coding errors may lead to either overlooked cases or overinterpretation of data, which could significantly impact the validity of conclusions [22]. The implications of diagnostic errors extend beyond mere statistical artifacts, as they may influence treatment pathways, compromise patient safety, and introduce variability into clinical datasets used for research and policy-making. These issues are not unique to clavicle fractures and could apply to a wide range of injuries. The relatively low incidence of medial fractures may contribute to their misclassification, as rare injuries are more prone to being overlooked, a common phenomenon documented in similar contexts [27,29,30,31,32]. Limited clinical exposure to these uncommon fractures may lead to increased diagnostic errors, both in radiological assessments and in the trauma specialties that manage these cases [31]. Additionally, linguistic similarities between the terms “medial” and “midshaft” may contribute to frequent mislabeling when meticulous attention to detail is lacking.

A lack of familiarity with ICD-10 classification guidelines, which categorize clavicle fractures by their location in thirds, may also play a role. Coding physicians or case managers who are unaware of this classification system may inadvertently mislabel diagnoses during the billing process [24,27]. Furthermore, the reliance on ambiguous or inconsistently applied coding systems can exacerbate these errors. Multiple or differing coding systems may be used in parallel, leading to discrepancies and misclassification during the documentation and billing processes. These potential inconsistencies underline the need for standardized coding practices and ongoing training for all involved coding/classifying personnel such as, for example, interdisciplinary case reviews or the use of standardized reporting templates.

### 4.3. Sources of Errors and Influence of Injury Complexity

The sources of diagnostic errors are multifactorial, with the most common errors occurring in billing codes, discharge summaries, and radiological reports. Our findings show that these errors often occur in multiple categories, indicating that mistakes may be perpetuated throughout the diagnostic and administrative process. For example, an error in the initial radiological diagnosis may be carried forward into the discharge summary and billing data. This conclusion is supported by our observation that errors in discharge summaries and billing codes frequently co-occur with errors in radiological reports, which are typically generated earlier in the care process.

The likelihood of diagnostic errors also increased with the number of imaging modalities used. Furthermore, more complex fractures, particularly those involving multiple regions of the clavicle, were associated with a higher probability of misclassification. This is understandable, as fractures that span multiple regions can be difficult to classify accurately using the existing ICD-10 system [24,33]. Multi-fragmentary clavicle fractures involving several parts of the clavicle may also be undercoded, as ICD-10 coding for such fractures (S42.09) is less frequently used in routine practice, despite being available [24].

These two factors—fracture complexity and the use of multiple imaging modalities—may be interconnected, as more complex fractures are often evaluated using more than one imaging technique. However, it remains unclear whether the increased error rate is due to the complexity of the injury itself or to additional factors such as associated injuries or the mechanism of trauma. Future studies should investigate whether the number of imaging modalities is an independent risk factor for diagnostic errors or simply a correlation of injury complexity.

Interestingly, our results showed that fewer diagnostic errors occurred with plain radiography compared to more advanced imaging techniques such as CT or MRI. This finding is counterintuitive, as one would expect greater diagnostic accuracy with more detailed imaging. A possible explanation is that plain radiographs provide a comprehensive view of the entire clavicle, which may be more helpful in classifying fractures than sectional imaging. Another factor could be the use of whole-body CT scans in polytrauma cases where the primary focus is on life-threatening injuries, potentially leading to diagnostic oversight for non-lethal (clavicle) fractures [34,35,36,37,38].

### 4.4. Limitations

This study has several limitations. First, it primarily focused on hospitalized cases, excluding conservatively treated outpatient cases. This may have introduced bias, as it is possible that less complex, conservatively managed cases are less prone to misclassification [3,39]. Additionally, the retrospective nature of the study precludes definitive causal conclusions. Retrospective analyses rely on pre-existing documentation, which may include incomplete, inconsistent, or inaccurate entries. These factors could systematically contribute to the observed diagnostic misclassifications as the clinical reasoning behind certain classification decisions cannot be reconstructed. It remains unclear which error category is the primary source of misclassification, and whether these errors were propagated over time or occurred simultaneously. It is more than challenging to determine whether these errors arose primarily from initial diagnostic assessments, subsequent documentation, or coding processes during administrative handling. Nevertheless, we believe that a retrospective design was more appropriate for this study, as a prospective study could introduce bias by raising awareness of the diagnostic coding process among healthcare providers. While our results shed light on the prevalence and potential sources of diagnostic errors in clavicle injury classification, definitive conclusions regarding their direct impact on patient outcomes remain speculative and would require further targeted investigation. Therefore, further prospective studies are necessary to validate and expand upon our findings.

A further limitation is the potential difficulty in distinguishing between lateral clavicle fractures and associated acromioclavicular joint dislocations. As lateral clavicle fractures could also occur accompanied by damage to the acromioclavicular ligaments, it may not always be appropriate to label the absence of a separate diagnosis of joint dislocation as an error. In practice, not all associated injuries are coded in the case of combined injuries or all regions of multi-fragmentary fractures, particularly if they are not revenue-relevant [27]. This raises the question of whether such omissions should be classified as errors, and what the true intention behind precise coding should be.

Finally, studies comparing Big Data analyses of routine data with clinically verified data would be valuable for assessing the impact of coding errors on large-scale data analytics, particularly in the era of AI-driven healthcare solutions [19]. Mislabeling can significantly limit the potential of algorithm-driven Big Data analyses, particularly those utilizing artificial intelligence [40,41]. The accuracy and benefit of these analyses are intrinsically tied to the quality of the underlying data [23,40,41]. Understanding the potential sources of misclassification and implementing routine, physician-led reviews of billing data may reduce the risk of bias and incorrect interpretation. This is particularly relevant in health services research and registry data analysis, where large datasets are integral to providing reliable, evidence-based insights [16,28,42]. While the determination of diagnostic errors in general, but also in terms of misclassification, is an important issue, further investigations are necessary concerning the topic of classification and coding accuracy in (clavicle) injury diagnoses.

## 5. Conclusions

The mislabeling of clavicle fractures is common, particularly between medial and midshaft fractures. Midshaft fractures are frequently misclassified as medial fractures, with errors occurring across diagnostic, administrative, and radiological categories. These errors increase with injury complexity and the use of multiple imaging techniques. Our study highlights a previously underexplored issue that warrants further investigation, as accurate diagnostic labeling is essential for reliable Big Data and AI-driven analyses. We emphasize the importance of considering the possibility and potential risks of erroneous classification. It is crucial that clinicians, researchers, and data analysts remain vigilant to these risks in order to avoid misclassifications. Misclassification can lead to erroneous conclusions and must be minimized to ensure the accuracy of future (Big Data) studies and clinical decision-making.

### Future Research

Future studies should explore the clinical and economic consequences of diagnostic misclassification. While the financial impact of coding errors may be minimal—since the same DRG is often billed for different clavicle fractures—the implications could be more significant in the context of hospital budget negotiations or insurance claims [7,27]. Misclassification could also affect statistical power in clinical research, as inaccurate diagnoses might lead to the over- or underestimation of case numbers.

Future research should also investigate whether the presence of accompanying injuries or the severity of trauma influences the likelihood of diagnostic errors. Further, studies should examine whether the mechanism of injury, insurance status, or treatment recommendations contribute to misclassification. Prospective studies would allow for the identification of when and where diagnostic errors occur in the care pathway, potentially leading to targeted interventions such as training for healthcare providers. It should focus on longitudinal, outcome-oriented studies to better quantify the direct effects of diagnostic errors on clinical endpoints and patient outcomes. Additionally, future research should explore whether similar rates of diagnostic misclassification are present in other types of injuries. We hope that this work will encourage future studies to adopt more robust classification methods and improve data accuracy across different medical contexts since it can have a significant impact on the quality and reliability of Big Data analyses and its interplay with artificial intelligence (AI) in diagnostic processes. On the other hand, AI-supported diagnostic systems could represent both a challenge and a promising solution to the issue of diagnostic misclassification. These systems have the potential to reduce human error, standardize diagnostic pathways, and improve overall data quality. We should explore the potential of AI-supported diagnostic systems not only as tools for error detection but also as proactive instruments to prevent misclassification. Integrating AI algorithms with Big Data analytics may enable real-time monitoring of diagnostic patterns, the early detection of anomalies, and the immediate correction of coding errors. However, their successful implementation relies on high-quality input data, highlighting the cyclical relationship between accurate classification and effective AI application.

In addition, studies should evaluate the robustness of AI systems when applied to datasets with pre-existing diagnostic inaccuracies. Understanding how these systems interact with flawed data is essential to prevent the perpetuation of biases and ensure the reliability of AI-supported diagnostics. Lastly, further studies should also explore how AI can assist in harmonizing diagnostic classifications across different healthcare systems and regions, ensuring consistency in coding practices on a global scale.

## Figures and Tables

**Table 1:** Distribution and epidemiological data of misclassified and correctly classified patients.

	Misclassified	Correctly Classified
Total Number of Patients *n*	379	1124
Percentage	25.2%	74.8%
Age (mean ± SD)	47.0 ± 18.3	44.9 ± 18.2
Sex male: *n* (%)	280 (73.9%)	853 (75.9%)
Sex female: *n* (%)	99 (26.1%)	271 (24.1%)

**Table 2:** Comparison of initial classification versus corrected classification. Numbers presented as percentage of all clavicle-related injuries.

Diagnosis Category	Initial Diagnosis (*n*)	Corrected Diagnosis (*n*)
Sternoclavicular Joint Dislocation (SCJD)	15 (0.81%)	14 (0.76%)
Medial Clavicle Fracture (MCF)	267 (14.39%)	96 (5.20%)
Midshaft Clavicle Fracture (MICF)	678 (36.55%)	886 (48.00%)
Lateral Clavicle Fracture (LCF)	491 (26.47%)	453 (24.54%)
Acromioclavicular Joint Dislocation (ACJD)	404 (21.78%)	397 (21.51%)

**Table 3:** A comparison of the Initial Fracture Diagnosis according to ICD 10 coding versus Corrected Coding and Actual Diagnosis of Clavicle Fractures (MCF, MICF, LCF). The numbers are presented as the percentage of all clavicle fractures. MCF = medial clavicle fracture; MICF = midshaft clavicle fracture; LCF = lateral clavicle fracture.

	Initial Fracture Diagnosis	Actual Fracture Diagnosis
MCF	267 (18.6%)	96 (6.7%)
MICF	678 (47.2%)	886 (61.7%)
LCF	491 (34.2%)	453 (31.6%)
Total	1436 (100.0%)	1435 (100.0%)

**Table 4:** Misdiagnosis rates by number of imaging modalities used. MRI deemed non-valid in this analysis as there were no cases where MRI was sole imaging modality used for diagnostic purposes.

Title 1	1× Imaging Modality			2× Imaging Modality			3× Imaging Modality
Imaging Modality	X-ray	CT	MRI	X-ray + CT	X-ray + MRI	CT + MRI	X-ray + CT + MRI
% Error Rate	17.96%	31.13%	Non-valid	32.09%	14.58%	0.00%	40.74%
% Total Error Rate	19.72%			30.54%			40.74%

## Data Availability

The data presented in this study are available within the manuscript.

## References

[1] Postacchini F., Gumina S., De Santis P., Albo F. (2002). Epidemiology of clavicle fractures. J. Shoulder Elb. Surg..

[2] Nordqvist A., Petersson C. (1994). The incidence of fractures of the clavicle. Clin. Orthop. Relat. Res..

[3] Bakir M.S., Unterkofler J., Haralambiev L., Kim S., Carbon R., Ekkernkamp A., Schulz-Drost S. (2020). Medial injuries of the clavicle: More prevalent than expected? A big data analysis of incidence, age, and gender distribution based on nationwide routine data. Eur. J. Trauma Emerg. Surg..

[4] Asadollahi S., Bucknill A. (2012). Acute medial clavicle fracture in adults: A systematic review of demographics, clinical features and treatment outcomes in 220 patients. J. Orthop. Traumatol..

[5] Ferree S., van Laarhoven J.J.E.M., Houwert R.M., Hietbrink F., Verleisdonk E.J.M.M., Leenen L.P.H. (2014). Distribution and treatment of clavicular fractures in monotrauma and polytrauma patients. J. Trauma Manag. Outcomes.

[6] Huttunen T.T., Launonen A.P., Berg H.E., Lepola V., Felländer-Tsai L., Mattila V.M. (2016). Trends in the Incidence of Clavicle Fractures and Surgical Repair in Sweden: 2001–2012. J. Bone Jt. Surg. Am. Vol..

[7] Wolf S., Chitnis A.S., Manoranjith A., Vanderkarr M., Plaza J.Q., Gador L.V., Holy C.E., Sparks C., Lambert S.M. (2022). Surgical treatment, complications, reoperations, and healthcare costs among patients with clavicle fracture in England. BMC Musculoskelet. Disord..

[8] Robinson C.M. (1998). Fractures of the clavicle in the adult. Epidemiology and classification. J. Bone Jt. Surg. Br. Vol..

[9] Khan L.A.K., Bradnock T.J., Scott C., Robinson C.M. (2009). Fractures of the Clavicle. J. Bone Jt. Surg..

[10] Bakir M.S., Unterkofler J., Hönning A., Haralambiev L., Kim S., Ekkernkamp A., Schulz-Drost S. (2019). Shoulder girdle injuries involving the medial clavicle differ from lateral clavicle injuries with a focus on concomitant injuries and management strategies: A retrospective study based on nationwide routine data. PLoS ONE.

[11] Bakir M.S., Merschin D., Unterkofler J., Guembel D., Langenbach A., Ekkernkamp A., Schulz-Drost S. (2017). Injuries of the Medial Clavicle: A Cohort Analysis in a Level-I-Trauma-Center. Concomitant Injuries. Management Classification. Chirurgia.

[12] Herteleer M., Winckelmans T., Hoekstra H., Nijs S. (2018). Epidemiology of clavicle fractures in a level 1 trauma center in Belgium. Eur. J. Trauma Emerg. Surg. Off. Publ. Eur. Trauma Soc..

[13] Kihlström C., Möller M., Lönn K., Wolf O. (2017). Clavicle fractures: Epidemiology, classification and treatment of 2 422 fractures in the Swedish Fracture Register; an observational study. BMC Musculoskelet. Disord..

[14] DGUV German Statutory Accident Insurance [Verletzungsartenverzeichnis mit Erläuterungen unter Einschluss des Schwerstverletzungsartenverfahrens]. DGUV Documents. Berlin, Germany. https://www.dguv.de/medien/landesverbaende/de/med_reha/documents/verletz3.pdf.

[15] Graydon C., Teede H., Sullivan C., Enticott J., De Silva K. (2022). Driving impact through big data utilization and analytics in the context of a Learning Health System. Driving Impact Through Big Data Utilization and Analytics in the Context of a Learning Health System.

[16] Djalali S., Markun S., Rosemann T. (2017). Routine Data in Health Services Research: An Underused Resource. Praxis.

[17] Gyftopoulos S., Lin D., Knoll F., Doshi A.M., Rodrigues T.C., Recht M.P. (2019). Artificial Intelligence in Musculoskeletal Imaging: Current Status and Future Directions. Am. J. Roentgenol..

[18] Prova O.S., Ahmed F., Sultana J., Ashrafuzzaman M., Keikhosrokiani P. (2022). Chapter 10—Big medical data analytics for diagnosis. Big Data Analytics for Healthcare.

[19] Biz C., Bragazzi N.L., Keikhosrokiani P. (2022). Chapter 22—Big data in orthopedics: Between hypes and hopes. Big Data Analytics for Healthcare.

[20] Wegscheider K., Koch-Gromus U. (2015). Die Versorgungsforschung als möglicher Profiteur von Big Data. Bundesgesundheitsblatt Gesundheitsforschung Gesundheitsschutz.

[21] Raghupathi W., Raghupathi V. (2014). Big data analytics in healthcare: Promise and potential. Health Inf. Sci. Syst..

[22] Meena T., Roy S. (2022). Bone Fracture Detection Using Deep Supervised Learning from Radiological Images: A Paradigm Shift. Diagnostics.

[23] Haserück A. (2021). Big Data: Datenmengen sinnvoll nutzen. Dtsch. Arztebl. Int..

[24] World Health Organization (2017). International Statistical Classification of Diseases and Related Health Problems.

[25] Ihaka R., Gentleman R. (1996). R: A Language for Data Analysis and Graphics. J. Comput. Graph. Stat..

[26] Filler B.C. (2007). Coding basics for orthopaedic surgeons. Clin. Orthop. Relat. Res..

[27] Klaus B., Ritter A., Grosse Hülsewiesche H., Beyrle B., Euler H.U., Fender H., Hübner M., von Mittelstaedt G. (2005). Study of the quality of codification of diagnoses and procedures under DRG conditions. Gesundheitswesen.

[28] Wang L., Alexander C.A. (2020). Big data analytics in medical engineering and healthcare: Methods, advances and challenges. J. Med. Eng. Technol..

[29] Knight A.W., Senior T.P. (2006). The common problem of rare disease in general practice. Med. J. Aust..

[30] Bakir M.S., Lefering R., Haralambiev L., Kim S., Ekkernkamp A., Gumbel D., Schulz-Drost S. (2020). Acromioclavicular and sternoclavicular joint dislocations indicate severe concomitant thoracic and upper extremity injuries in severely injured patients. Sci. Rep..

[31] Lin Y.K., Lin C.J., Chan H.M., Lee W.C., Chen C.W., Lin H.L., Kuo L.C., Cheng Y.C. (2014). Surgeon commitment to trauma care decreases missed injuries. Injury.

[32] Keijzers G.B., Giannakopoulos G.F., Del Mar C., Bakker F.C., Geeraedts L.M. (2012). The effect of tertiary surveys on missed injuries in trauma: A systematic review. Scand. J. Trauma Resusc. Emerg. Med..

[33] (2013). Federal Ministry of Justice and Consumer Protection [Bundesministerium der Justiz und für Verbraucherschutz]. Hospital Remuneration Act [Krankenhausentgeltgesetz], 860–5–24.§. Laws on the Internet [Gesetze im Internet]. www.gesetze-im-internet.de/khentgg/__4.html.

[34] Zamboni C., Yonamine A.M., Faria C.E., Filho M.A., Christian R.W., Mercadante M.T. (2014). Tertiary survey in trauma patients: Avoiding neglected injuries. Injury.

[35] Pfeifer R., Pape H.C. (2008). Missed injuries in trauma patients: A literature review. Patient Saf. Surg..

[36] Horst K., Hildebrand F., Kobbe P., Pfeifer R., Lichte P., Andruszkow H., Lefering R., Pape H.C. (2015). Detecting severe injuries of the upper body in multiple trauma patients. J. Surg. Res..

[37] Stevens N.M., Tejwani N. (2018). Commonly Missed Injuries in the Patient with Polytrauma and the Orthopaedist’s Role in the Tertiary Survey. J. Bone Jt. Surg. Rev..

[38] Scaglione M., Iaselli F., Sica G., Feragalli B., Nicola R. (2015). Errors in imaging of traumatic injuries. Abdom. Imaging.

[39] Herteleer M., Hoekstra H., Nijs S. (2018). Diagnosis and treatment of clavicular fractures in Belgium between 2006 and 2015. J. Shoulder Elb. Surg..

[40] Reichardt A. (2021). Künstliche Intelligenz in der Medizin: Lernen im Schwarm. Dtsch. Arztebl. Int..

[41] Krüger-Brand H.E. (2015). Big Data und Gesundheit: Viele Hoffnungen, viele Ängste. Dtsch. Arztebl. Int..

[42] Stengel D., Mutschler W., Dubs L., Kirschner S., Renkawitz T. (2021). Klinische Studien in Unfallchirurgie und Orthopädie: Lesen, interpretieren und umsetzen. Unfallchirurg.

